# Aberrant Functional Connectivity Architecture in Alzheimer's Disease and Mild Cognitive Impairment: A Whole-Brain, Data-Driven Analysis

**DOI:** 10.1155/2015/495375

**Published:** 2015-06-08

**Authors:** Bo Zhou, Hongxiang Yao, Pan Wang, Zengqiang Zhang, Yafeng Zhan, Jianhua Ma, Kaibin Xu, Luning Wang, Ningyu An, Yong Liu, Xi Zhang

**Affiliations:** ^1^Department of Neurology, Institute of Geriatrics and Gerontology, Chinese PLA General Hospital, Beijing 100853, China; ^2^Department of Radiology, Chinese PLA General Hospital, Beijing 100853, China; ^3^Hainan Branch of Chinese PLA General Hospital, Sanya 572014, China; ^4^Brainnetome Center, Institute of Automation, Chinese Academy of Sciences, Beijing 100190, China; ^5^School of Biomedical Engineering, Southern Medical University, Guangzhou, Guangdong 510515, China; ^6^National Laboratory of Pattern Recognition, Institute of Automation, Chinese Academy of Sciences, Beijing 100190, China

## Abstract

The purpose of our study was to investigate whether the whole-brain functional connectivity pattern exhibits disease severity-related alterations in patients with Alzheimer's disease (AD) and mild cognitive impairment (MCI). Resting-state functional magnetic resonance imaging data were acquired in 27 MCI subjects, 35 AD patients, and 27 age- and gender-matched subjects with normal cognition (NC). Interregional functional connectivity was assessed based on a predefined template which parcellated the brain into 90 regions. Altered whole-brain functional connectivity patterns were identified via connectivity comparisons between the AD and NC subjects. Finally, the relationship between functional connectivity strength and cognitive ability according to the mini-mental state examination (MMSE) was evaluated in the MCI and AD groups. Compared with the NC group, the AD group exhibited decreased functional connectivities throughout the brain. The most significantly affected regions included several important nodes of the default mode network and the temporal lobe. Moreover, changes in functional connectivity strength exhibited significant associations with disease severity-related alterations in the AD and MCI groups. The present study provides novel evidence and will facilitate meta-analysis of whole-brain analyses in AD and MCI, which will be critical to better understand the neural basis of AD.

## 1. Introduction

It has been estimated that more than 81.1 million individuals will suffer from dementia by 2040, and Alzheimer's disease (AD) will account for the underlying pathology in the majority of these cases [1]. Mild cognitive impairment (MCI) is a stage involving greater cognitive decline than expected based on an individual's age and educational level. MCI is thought to be the prodromal stage of dementia; in particular, the amnestic subtype of MCI carries a very high risk of progression to AD [2]. Nevertheless, the definitive relationship between AD and MCI requires further investigation.

The past decade has witnessed great progress in resting-state functional magnetic resonance imaging (rs-fMRI), which is based on the measurement of spontaneous low-frequency fluctuations of blood oxygen level-dependent (BOLD) signals [3]. The correlations/similarities of these types of fluctuations among various brain regions have been thought to represent the interregional functional connectivity [4]. Convergent evidence identified via rs-fMRI has suggested that alterations in functional connectivity/networks are prevalent in AD and MCI [5–14]. Thus, the previous literature has suggested that AD/MCI is a disconnection syndrome [15–17].

Despite previous elegant studies that identified alterations in the connections between specific brain regions or networks [8, 10, 18–23], the patterns of whole-brain resting-state functional connectivity in AD and MCI have not been well studied, which may limit our understanding of the pathophysiological substrate of the disease from an integrative perspective. In the first whole-brain connectivity study in AD, Wang and colleagues demonstrated that AD patients exhibited an anterior-posterior disconnection phenomenon, especially between the prefrontal and parietal lobes, as well as compensatory increases in intralobe connections [11]. In MCI subjects, Bai and colleagues also found diffuse abnormalities in functional connections, especially between the subcortical regions and the frontal cortices. These disturbances were related to cognitive variables and became more evident over time [5]. Almost at the same time, Chen and colleagues demonstrated that impairments in the functional connectivity strength were significantly correlated with cognitive abilities in AD/MCI subjects, and the large-scale interconnectivity patterns among brain regions can be used to differentiate cognitively normal subjects from patients with AD or MCI [7]; these findings are consistent with other independent classification studies [24–26]. Using detailed parcellated brain regions, Liu and colleagues demonstrated that the disease severity was related to the loss of long-distance connectivity in AD and MCI [9]. Despite the diversity of the results, these studies support the hypothesis that AD is a disconnection syndrome [16, 17]. Moreover, an additional independent whole-brain functional connectivity study based on a larger sample will further strengthen our understanding of the impaired functional connectivity patterns and provide novel evidence for a future meta-analysis of AD/MCI.

**Figure 1:**
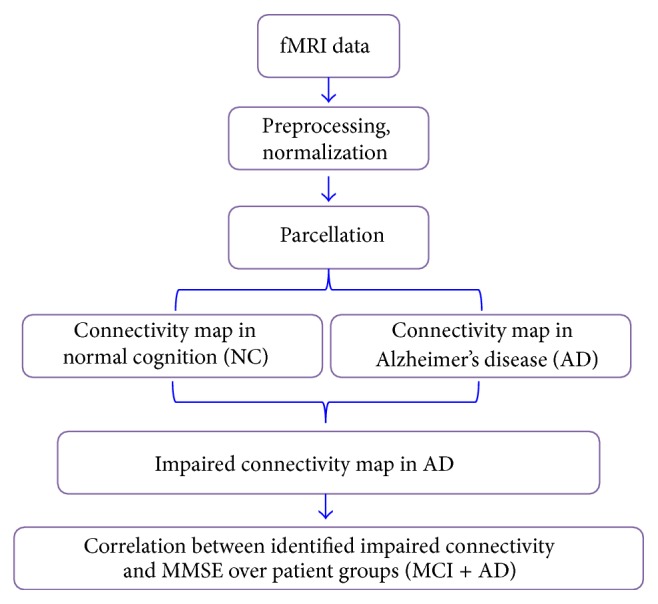
Schematic map of the experimental design of the present study.

Based on the previous literature, we hypothesized that the changes in functional connectivity would represent the distribution of decreased long-distance interregions in AD. We also expected that the altered brain functional connectivity in AD patients would be decreased in subjects with MCI. Furthermore, the abnormal brain functional connectivity would be correlated with variations between patients in the severity of cognitive impairment according to the mini-mental state examination (MMSE). To test these hypotheses in the current study, we explored functional connectivity throughout the brain to investigate whether alterations exist in 35 patients with severe AD and 27 age-matched volunteers with normal cognition (NC). First, we investigated interregional functional connectivity by dividing each individual's brain into 90 regions using an automated anatomical labeling (AAL) template [27]. Second, we identified significant differences in functional connectivities via comparisons of the correlation coefficients of each pair of brain regions between the AD and NC samples. To determine whether the identified altered functional connectivity varied with disease progression, Pearson's correlation analyses were performed between the functional connectivity strengths and the clinical variables (MMSE) in the MCI and AD patients (Figure 1).

## 2. Materials and Methods

The samples used in the present study have been examined in our previous studies of regional homogeneity [13], amygdalar connectivity [12, 28], thalamic connectivity [14], and marginal division connectivity [29] during resting states. All subjects met identical methodological stringency criteria, and the comprehensive clinical details have been described in our previous work [12–14, 29]. This study is orthogonal to any previously published studies, which ensures the independence of the reported effects. However, we have provided a brief introduction regarding the data inclusion/exclusion criteria, acquisition, and processing to maintain the scientific integrity of the present study.

### 2.1. Standard Protocol Approvals, Registration, and Patient Consent

This study was approved by the Medical Ethics Committee of PLA General Hospital. Written informed consent was obtained from each enrolled subject or his/her authorized guardian. The participants underwent general physical, psychological, and laboratory examinations prior to enrollment in the formal study. The participants did not take medications that might have influenced cognition during the scans, and all patients received professional suggestions for further treatment.

### 2.2. Subjects

The participants were recruited from two sources: outpatients from the Chinese PLA General Hospital or recruitment through a website advertisement (http://www.301ad.com.cn/, Chinese version). Each subject was right-handed and underwent a battery of neuropsychological tests, including the MMSE, Montreal Cognitive Assessment, Trail Making Test, Clock Drawing Test, Similarities Test, Complex Figure Replication, Verbal Fluency Test, Auditory Verbal Learning Test (AVLT), Geriatric Depression Scale, Clinical Dementia Rating (CDR), and Activities of Daily Living (ADL) scale. The detailed diagnostic criteria for AD, amnestic MCI, and normal healthy aging can be found in our previous studies [12–14]. Briefly, following the exclusion of subjects with substantial head motion (see the criteria for data preprocessing), 89 subjects, including 27 MCI subjects, 35 AD patients, and 27 age- and gender-matched normal cognitive (NC) subjects, were included in the subsequent analyses. The demographic and neuropsychological details regarding the subjects are shown in Table S1 (see Table S1 in Supplementary Material available online at http://dx.doi.org/10.1155/2015/495375).

### 2.3. Data Acquisition

A 3.0 T GE MR system (GE Healthcare, USA) with a standard head coil was used to acquire the MR images. Resting-state fMRI scans were performed using an echo planar imaging (EPI) sequence with the following parameters: repetition time = 2,000 ms, echo time = 30 ms, flip angle = 90°, matrix = 64 × 64, field of view = 220 × 220 mm^2^, slice thickness = 3 mm, and slice gap = 1 mm. Each volume comprised 30 axial slices, and each functional run lasted for 6 minutes and 40 seconds. During the scanning, the subjects were instructed to keep their eyes closed and to relax; comfortable foam padding was used to minimize head motion, and ear plugs were used to reduce the scanner noise. For each subject, T2-weighted images were collected and evaluated by two senior radiologists during the scan.

### 2.4. Connectivity Analysis Pipeline

The analysis consisted of the following steps: (1) data preprocessing; (2) defining brain nodes with whole-brain parcellation; (3) calculating connectivity matrices for each subject; (4) comparing matrices to identify significant differences between groups in terms of correlation strength; and (5) investigating the relationships between altered functional connectivity and cognitive variables (see Figure 1 for a schematic of this analysis).

### 2.5. Data Preprocessing

The data were preprocessed in steps consistent with the protocols of our previously published studies using in-house Brainnetome fMRI toolkit (Brat, http://www.brainnetome.org/en/brat) based on statistical parametric mapping (SPM8, http://www.fil.ion.ucl.ac.uk/spm/). These steps included (1) slice-timing for time correction, (2) realignment to reduce head motion, (3) normalization to a standard EPI template and reslicing to 2 × 2 × 2 mm cubic voxels, (4) denoising by regressing out several effects, for example, six motion parameters, linear drift, and the mean time series of all voxels within the white matter and cerebrospinal fluid, and (5) temporal filtering (0.01–0.08 Hz) to reduce noise [9, 12–14].

### 2.6. Defining the Connectivity Nodes

The registered normalized fMRI time series were segmented into 90 regions (45 regions per hemisphere, Table S2) using an automated anatomical labeling template [27] that has been used in several previous studies [30–36]. For each sample, a representative time series of each brain region was obtained by averaging the fMRI time series over all voxels in the region.

### 2.7. Estimation of Interregional Functional Connectivity

The regional mean time series were estimated by averaging the time series of all voxels in the region [31, 37, 38]. Pearson's correlation coefficients were computed between each pair of brain regions for each subject. For subsequent statistical analysis, Fisher's *r*-to-*z* transformation was applied to improve the normality of the correlation coefficients [32, 33]. To determine whether disease severity-related alterations existed, we also evaluated altered whole-brain connectivity patterns between the NC and MCI, MCI and AD, and NC and AD groups.

Individual *z*-scores were compared using a one-sample two-tailed *t*-test to determine whether the two brain regions exhibited significant functional connectivity within each group. They were also analyzed by a two-sample two-tailed *t*-test to determine whether the functional connectivities were significantly different between the AD and NC groups. A two-sample two-tailed *t*-test was performed for all 4005 (90 × 89/2) functional connectivities; thus, a correction for multiple comparisons was strictly necessary. The false discovery rate (FDR) approach was applied to identify a threshold that would restrict the expected proportion of type I errors [38, 39]. In this study, we identified significant differences in the functional connectivities between the normal healthy and AD subjects according to the following two criteria: (a) the *z* values were significantly different from zero in at least one group at *P* < 0.05 (one-sample two-tailed *t*-test; Bonferroni corrected) and (b) the *z*-scores were significantly different between the two groups at *P* < 0.05 (two-sample two-tailed *t*-test; FDR-corrected).

### 2.8. Relationship between Altered Functional Connectivity and Cognitive Ability

To investigate the relationship between functional connectivity strength and cognitive ability, we also evaluated Pearson's correlation between the MMSE scores (as a measure of cognitive function) and functional connectivity strength among the identified functional connectivities in the MCI, AD, and MCI plus AD groups. Because these relationships were exploratory in nature, we used a statistical significance level of *P* < 0.05 (uncorrected).

## 3. Results

### 3.1. Direct Comparisons between Groups

**Figure 2:**
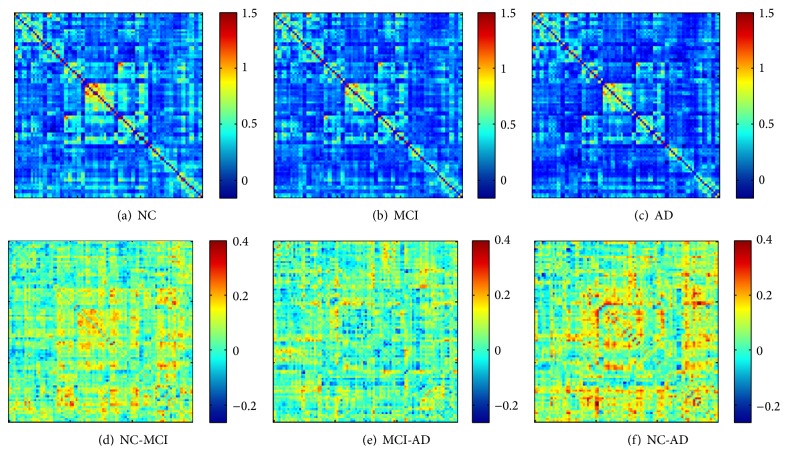
Mean absolute *z*-score matrices for normal control (a), MCI (b), and AD (c). Each figure shows a 90 × 90 square matrix, in which each entry indicates the mean functional connectivity strength between the corresponding pair of brain regions. The diagonal running from the lower right to the upper left is intentionally set in black. The *z*-score of the functional connectivity is indicated with a colored bar. The lower row indicates the regions that exhibit visual differences between the normal control and MCI (d), MCI and AD (e), and normal control and AD groups (f), which were calculated using the functional connectivity strengths of the former minus the latter.

For each group, the mean functional connectivity matrix was calculated by averaging the *N* × *N* (*N* = 90 in the present study) connection matrix of all subjects. In the NC group, most of the strong functional connectivities (large *z*-scores) were between interhemispheric homogeneous regions, within a lobe, and between anatomically adjacent brain areas (Figure 2(a)), which is consistent with many previous studies of whole-brain functional connectivity during the resting state [9, 11, 30–32, 36, 40]. The AD and MCI groups exhibited similar functional connectivity patterns compared with that of the NC group (main effect of group, *F*_2,86_ = 2.55, *P* = 0.084) (Figures 2(b) and 2(c)). Post hoc analysis demonstrated that the mean correlation strength was slightly lower in the AD group compared with the normal cognitive subjects (main effect of group, *F*_1,52_ = 3.4, *P* = 0.070). The mean correlation strength in the MCI group was located between those of the normal cognitive and AD groups (Figures 2(d)–2(f)).

**Figure 3:**
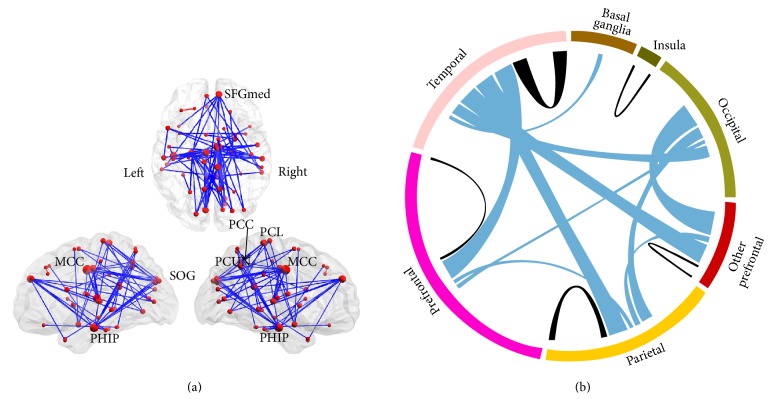
Altered whole-brain connectivity patterns in the AD group compared with the normal control group. (a) Three-dimensional representation of the connectivities and most of the affected nodes (*P* < 0.05, FDR-corrected) in AD. The blue and red lines denote decreased and increased functional connectivities, respectively. (b) Distribution of the altered functional connectivities. The colored ring represents the various brain lobes. The blue and black colors represent the interlobe and intralobe functional connectivities, respectively. For details, please see Tables S2-S3 and Figure S1.

Specifically, compared with the normal cognitive subjects, the AD group exhibited decreased functional connectivities at the threshold of *P* < 0.05 (FDR-corrected) (Figure 3(a)). The most significantly affected regions included several important nodes of the default mode network (DMN), such as the posterior cingulate gyrus (PCC), the medial superior frontal gyrus (SFGmed), the precuneus (PCUN), and the parahippocampal gyrus (PHIP), as well as the median- and paracingulate gyrus (MCC), the superior occipital gyrus (SOG), and the paracentral lobule (PCL) (Figure 3(a), for details, please refer to Table S3 and Figure S1). We also noted that the most affected type of functional connectivity was the interlobe connections, such as the temporal lobe to the frontal and parietal lobes, and that the most affected brain lobe was the temporal lobe (Figure 3(b), for details, please refer to Table S3 and Figure S1).

### 3.2. Clinical Cognitive Variables and Functional Connectivity Strength

**Figure 4:**
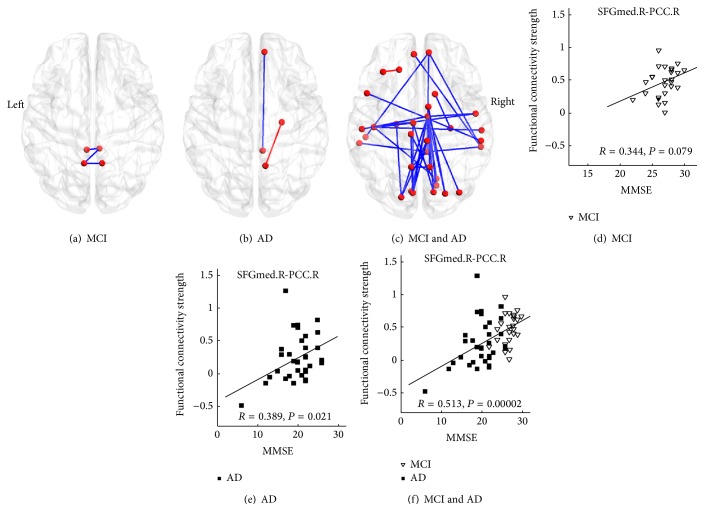
The correlation between the MMSE scores and the functional connectivity strengths. The upper line denotes the functional connectivities significantly correlated with the MMSE scores in the MCI (a), AD (b), and combined MCI and AD (c) groups. The blue color represents the functional connectivity that is positively correlated with the MMSE scores, and the red color represents the functional connectivity that is negatively correlated with the MMSE scores. The lower line denotes the correlation between the MMSE scores and the functional connectivity strength (e.g., between the right medial superior frontal gyrus (SFGmed) and the posterior cingulate gyrus (PCC)) in the MCI (d), AD (e), and combined AD and MCI (f) groups. For details, please refer to Table S3.

The results showed that approximately half (35 of 76 altered connectivities) of the decreased functional connectivities exhibited significantly positive correlations with the MMSE scores in the MCI and AD patients (*P* < 0.05). Namely, increased illness severity was correlated with reduced functional connectivity strength (Figure 4(c), Table S3). For the identified altered brain regions, we determined that only a subset of functional connectivities between the various regions were significantly correlated with the MMSE scores in the AD or MCI groups (Figures 4(a) and 4(b) and Table S3). For example, the functional connectivity between the right medial superior frontal gyrus (SFGmed) and the posterior cingulate gyrus (PCC) exhibited the strongest correlation in the patients (Figure 4(f) and Table S3); there was also a strong correlation in the AD group (Figure 4(e)) and a tendency toward correlation in the MCI group (Figure 4(d)).

## 4. Discussion

Consistent with previous studies, the present study identified widespread impaired functional connectivity patterns in AD patients, including anterior-posterior and interlobe disconnections [5, 7, 9, 11, 25]. These findings indicate that the pattern of decreased long-distance connection is a consistent functional manifestation in AD patients and supports the notion that AD is a disconnection syndrome [16, 17]. More importantly, the present results demonstrated that the identified impaired connectivity strengths in the MCI patients were located between those of the normal cognitive subjects and AD patients (Figures 2(d)–2(f)) and that most of the identified functional connectivity strengths were significantly correlated with cognitive variables in the patient groups (Figure 4). Thus, these findings provide additional evidence that MCI is a prodromal stage of AD [41].

Consistent with the findings of previous whole-brain studies [11, 26], the present study demonstrated that the connectivities of several important nodes of the default mode network, such as the PCC, the precuneus, the parahippocampal gyrus, and the medial superior frontal gyrus, are affected in AD/MCI subjects (Figures 4 and S1 and Table S2). The default mode network plays a key role in various cognitive processes, such as remembering past events, envisioning the future [3, 42–44], and episodic memory [6, 43]. These cognitive functions are particularly vulnerable in AD/MCI [45, 46] and have been thoroughly studied using multiple imaging techniques, including positron emission tomography, diffusion MRI, structural MRI, and functional MRI. Imaging findings have consistently identified abnormal changes in the default mode network and its relationship to the cognitive degradation observed in AD/MCI patients (for a review, see [6, 8, 47–49]). It should be highlighted that the present study demonstrated that decreased functional connectivity is positively correlated with impaired cognitive ability according to MMSE scores (Figure 4 and Table S3). This finding illustrates that the default mode network is the most affected network, and an abnormal change in this network may represent a potential biomarker for the early identification of MCI and AD.

The temporal lobe, especially the parahippocampal gyrus, exhibited most changes in interlobe functional connectivity (Figures 3 and S1). The temporal lobe is associated with complex functions that range from primary auditory sensation to advanced cognitive roles, such as social cognition and memory [50–52], and most of these functions are impaired in AD/MCI. The anterior parahippocampal region was identified as the first site of neurofibrillary tangles in AD via neuropathological studies [53, 54]. Previous studies have also indicated that volume loss of parahippocampal white matter contributes to the memory impairments observed in mild AD and can be considered a predictor of MCI and AD [55, 56]. Studies have also indicated that certain regions in the temporal lobe, especially the parahippocampus and hippocampus, are key regions of the episodic memory network [57–59]. Episodic memory impairment is typically the earliest symptom and a core clinical symptom of AD and MCI [45, 46]. Therefore, the impaired interlobe functional connectivity of the temporal lobe and its correlation with reduced cognitive ability may be indirectly associated with broad cognitive functional degradation, such as the episodic memory impairment in AD/MCI subjects.

We also found abnormal functional connectivities to the MCC in the AD subjects in the present study (Figures 3 and S1 and Table S3), in agreement with the results of previous studies [5, 11]. The MCC is involved in rather complex cognitive and emotional functions (e.g., cognitive control and negative affect) [60–63] and has been identified as one of the most affected regions by the impaired glucose metabolism that occurs in AD/MCI [64]. Interestingly, the connectivity strengths between the MCC and several regions, including the precuneus and superior temporal lobe, were significantly correlated with the MMSE scores in the AD and MCI groups (Figure 4(c), Table S3). Considering this phenotype and the fact that emotional dysfunction is also a key component of clinical manifestations of AD/MCI [65], we speculate that the altered functional connections between the MCC and other regions may be related to cognitive and emotional impairments in AD/MCI.

It should be noted that we assessed whole-brain functional connectivity to evaluate disease severity-related altered functional connectivity patterns in AD subjects. We then investigated the patterns of the identified connectivities in MCI, which may have missed some information, such as the connectivities that exhibited a compensatory increase in the AD/MCI subjects. The identified altered functional connectivities in AD did not exhibit strong correlations with those of the MCI group regarding the relationship between functional connectivity strength and cognitive ability; only the combined patient groups exhibited significant disease severity-related correlations. One potential reason may be because of the limited sample size of each subgroup; thus, these findings should be carefully interpreted because not all MCI subjects will convert to AD. We should also note that the atlas used in the present study is still not a well-defined parcellated template for regions with inhomogeneous functions; for example, the thalamus can be further parcellated into finely defined subregions [66, 67]. Thus, the development of a new, well-defined connectivity atlas combined with the assessment of neuropsychiatric symptoms is needed to better elucidate the precise neural mechanism of AD/MCI.

## 5. Conclusion

The present data-driven whole-brain functional connectivity study demonstrated that brain functional connectivity patterns are significantly impaired in AD/MCI patients. Distributions of abnormal functional connectivity were identified in several important nodes of the default mode network, but they were not confined to this network. More importantly, decreased functional connectivity strength was significantly and positively correlated with the MMSE scores in the MCI and AD patients, suggesting that altered connectivities are related to disease severity and clinical manifestations. These results increase the current understanding of the specific alterations in whole-brain functional connectivity patterns in these patients and contribute novel data for future meta-analysis to examine the impaired connectivity patterns in AD/MCI.

## Supplementary Material

Table S1: Demographic, clinical and neuropsychological data in normal control (NC), mild cognitive impairment (MCI) and Alzheimer's disease (AD) subjects.
Table S2: Cortical and subcortical regions defined in Automated Anatomical Labeling template image in standard stereotaxic space.
Table S3: The impaired connectivity AD, also the correlation between MMSE and strength of the functional connectivity.
Figure S1: Distributed of the impaired functional connectivity in AD. Each line indicates the mean strength of the functional connectivity between each pair of brain regions. Blue lines represent inter connectivity between lobs and grey lines mean intra connectivity within the defined lobes in Table S2. Detail for these connectivity and it's related to MMSE can be found at Table S3 and Figure 3.
