# Supplementary material for: Aberrant Functional Connectivity Architecture in Alzheimer's Disease and Mild Cognitive Impairment: A Whole-Brain, Data-Driven Analysis
Source: Biomed Res Int. 2015 Jun 8;2015:495375. doi: 10.1155/2015/495375 (PMC4475740; doi:10.1155/2015/495375)
Supplement: Supplementary file 1 — Table S1: Demographic, clinical and neuropsychological data in normal control (NC), mild cognitive impairment (MCI) and Alzheimer's disease (AD) subjects. Table S2: Cortical and subcortical regions defined in Automated Anatomical Labeling template image in standard stereotaxic space. Table S3: The impaired connectivity AD, also the correlation between MMSE and strength of the functional connectivity. Figure S1: Distributed of the impaired functional connectivity in AD. Each line indicates the mean strength of the functional connectivity between each pair of brain regions. Blue lines represent inter connectivity between lobs and grey lines mean intra connectivity within the defined lobes in Table S2. Detail for these connectivity and it's related to MMSE can be found at Table S3 and Figure 3. [file 495375.f1.pdf]

Aberrant functional connectivity architecture in Alzheimer's disease and mild cognitive impairment: A whole-brain, data-driven analysis

Bo Zhou[1], Hongxiang Yao[2], Pan Wang[1], Zengqiang Zhang[1,3], Yafeng Zhan[4,5], Jianhua Ma[5], Kaibin Xu[4,6], Luning Wang[1], Ningyu An[2], Yong Liu[4, 6, CA], Xi Zhang[1, CA]

[1]Department of Neurology, Institute of Geriatrics and Gerontology, Chinese PLA General Hospital, Beijing, 100853, China

[2]Department of Radiology, Chinese PLA General Hospital, Beijing, 100853, China

[3]Hainan Branch of Chinese PLA General Hospital, Sanya, 572014, China

[4]Brainnetome Center, Institute of Automation, Chinese Academy of Sciences, Beijing, 100190, China

[5]School of Biomedical Engineering, Southern Medical University, Guangzhou, Guangdong, 510515, China

[6]National Laboratory of Pattern Recognition, Institute of Automation, Chinese Academy of Sciences, Beijing, 100190, China

Correspondence to

Dr. Xi Zhang

Department of Neurology, Institute of Geriatrics and Gerontology, Chinese PLA General Hospital, Beijing, 100853, China

E-mail: zhangxi@301hospital.com.cn
Dr. Yong Liu
Brainnetome Center, Institute of Automation, Chinese Academy of Sciences, Beijing, 100190, China
Email: yliu@nlpr.ia.ac.cn

Outline

Table S1. 3

Table S2. 4

Table S3 6

Figure S1..10

Reference 11

Table S1.

Table S1. Demographic, clinical and neuropsychological data in normal control (NC), mild cognitive impairment (MCI) and Alzheimer's disease (AD) subjects.

	NC(n=27)	MCI(n=27)	AD(n=35)	p value
Gender (M/F)	16/11	13/14	12/23	0.143
Age (year)	69.2±6.5	73.8±7.8	72.4±8.5	0.09
MMSE	28.9±1.0	26.8±1.8[a]	19.7±4.1 [a,b]	<0.001
CDR	0	0.5	1.3±0.5 [a,b]	<0.001
AVLT-Immediate Recall [c]	5.9±1.1	4.6±1.5	2.6±1.6 [a,b]	<0.001
AVLT-Delay Recall [c]	5.8±2.0	3.1±2.0 [a]	0.6±1.2 [a,b]	<0.001
Head Motion	0.25±0.27	0.16±0.10	0.30±0.27	0.084

Chi-square was used for gender comparisons, One-way ANOVA with Bonferroni post hoc test was used for age, and neuropsychological tests comparisons

[a]Significant compared to NC. [b] Significant compared to MCI.[c] Three AD subjects refuse to continue this test.

MMSE, mini-mental state examination; CDR, Clinical Dementia Rating; AVLT, auditory verbal learning test.

Note: For all the data was the same with our previous studies(Zhou et al., 2013, Zhang et al., 2014), hence the data information is the same with those papers.

Table S2.

Table S2. Cortical and subcortical regions defined in Automated Anatomical Labeling template image in standard stereotaxic space

Region Name	Abbreviation	MNI coordinates (L/R)
**Prefrontal Lobe**		(-18,35,42)/ (22,31,44)
Superior frontal gyrus, dorsolateral	SFGdor	(-17,47,-13)/ (18,48,-14)
Superior frontal gyrus, orbital	SFGorb	(-5,49,31)/ (9,51,30)
Superior frontal gyrus, medial	SFGmed	(-5,54,-7)/ (8,52,-7)
Superior frontal gyrus, medial orbital	SFGmorb	(-33,33,35)/ (38,33,34)
Middle frontal gyrus	MFG	(-31,50,-10)/ (33,53,-11)
Middle frontal gyrus, orbital	MFGorb	(-48,13,19)/ (50,15,21)
Inferior frontal gyrus, opercular	IFGoper	(-46,30,14)/ (50,30,14)
Inferior frontal gyrus, triangular	IFGtri	(-36,31,-12)/ (41,32,-12)
Inferior frontal gyrus, orbital	IFGorb	(-5,37,-18)/ (8,36,-18)
Gyrus rectus	REG	(-4,35,14)/ (8,37,16)
Anterior cingulate gyrus	ACC	(-8,15,-11)/ (10,16,-11)
Olfactory cortex	OLF	(-18,35,42)/ (22,31,44)
**Other Prefrontal Lobe**		
Precentral gyrus	PreCG	(-39,-6,51)/ (41,-8,52)
Supplementary motor area	SMA	(-5,5,61)/ (9,0,62)
Rolandic operculum	ROL	(-47,-8,14)/ (53,-6,15)
Median- and para-cingulate gyrus	MCC	(-5,-15,42)/ (8,-9,40)
**Occipital Lobe**		
Calcarine fissure and surrounding cortex	CAL	(-7,-79,6)/ (16,-73,9)
Cuneus	CUN	(-6,-80,27)/ (14,-79,28)
Lingual gyrus	LING	(-15,-68,-5)/ (16,-67,-4)
Superior occipital gyrus	SOG	(-17,-84,28)/ (24,-81,31)
Middle occipital gyrus	MOG	(-32,-81,16)/ (37,-80,19)
Inferior occipital gyrus	IOG	(-36,-78,-8)/ (38,-82,-8)
Fusiform gyrus	FG	(-31,-40,-20)/ (34,-39,-20)
**Parietal Lobe**		
Superior parietal gyrus	SPG	(-23,-60,59)/ (26,-59,62)
Paracentral lobule	PCL	(-7,-56,48)/ (10,-56,44)
Postcentral gyrus	PoCG	(-42,-23,49)/ (41,-25,53)
Inferior parietal gyrus	IPG	(-43,-46,47)/ (46,-46,50)
Supramarginal gyrus	SMG	(-56,-34,30)/ (58,-32,34)
Angular gyrus	ANG	(-44,-61,36)/ (46,-60,39)
Precuneus	PCNU	(-8,-25,70)/ (7,-32,68)
Posterior cingulate gyrus	PCC	(-5,-43,25)/ (7,-42,22)
**Insula**	INS	(-35,7,3)/ (39,6,2)

**Thalamus**	THA	(-11,-18,8)/ (13,-18,8)
**Temporal Lobe**		
Superior temporal gyrus	STG	(-53,-21,7)/ (58,-22,7)
Superior temporal gyrus, temporal pole	STGp	(-40,15,-20)/ (48,15,-17)
Middle temporal gyrus	MTG	(-56,-34,-2)/ (57,-37,-1)
Middle temporal gyrus, temporal pole	MTGp	(-36,15,-34)/ (44,15,-32)
Inferior temporal gyrus	ITG	(-50,-28,-23)/ (54,-31,-22)
Heschl gyrus	HES	(-42,-19,10)/ (46,-17,10)
Hippocampus	HIP	(-25,-21,-10)/ (29,-20,-10)
Parahippocampal gyrus	PHIP	(-21,-16,-21)/ (25,-15,-20)
Amygdala	AMYG	(-23,-1,-17)/ (27,1,-18)
**Basal Ganglia**		
Caudate nucleus	CAU	(-11,11,9)/ (15,12,9)
Lenticular nucleus, putamen	PUT	(-24,4,2)/ (28,5,2)
Lenticular nucleus, pallidum	PAL	(-18,0,0)/ (21,0,0)

Note: The abbreviations listed are those used in this paper, which differ slightly from the original abbreviations by Tzourio-Mazoyer et al. (2002). And the center of mass coordinates are the same with Liu et al. (2007).

MNI: Montreal Neurological Institute.

Table S3

Table S3, The impaired connectivity AD, also the correlation between MMSE and strength of the functional connectivity.

Node_1	Node_2	P	NC_mean	NC_sd	MCI mean	MCI sd	AD mean	AD sd	MMSE MC_ZR	MMSE MC_P	MMSE AD_ZR	MMSE AD_P	MMSE MC_AD_ZR	MMSE MC_AD_P	Type
SFGmed.R	PCC.R	0.00042	0.538	0.284	0.472	0.226	0.229	0.351	0.344	0.079	0.389	0.021	**0.513**	**0.000**	PreF-P
MCC.R	MOG.R	0.00076	0.403	0.291	0.384	0.249	0.135	0.298	0.248	0.212	0.189	0.276	**0.421**	**0.001**	OthF-O
MCC.R	CAL.R	0.00011	0.339	0.273	0.271	0.233	0.017	0.324	0.230	0.248	0.177	0.310	**0.410**	**0.001**	OthF-O
PCC.R	MTG.R	0.00005	0.471	0.278	0.409	0.208	0.167	0.264	0.144	0.472	0.132	0.448	**0.408**	**0.001**	P-T
MCC.R	CUN.R	0.00006	0.459	0.257	0.394	0.213	0.153	0.291	0.296	0.134	0.121	0.489	**0.402**	**0.001**	OthF-O
PCC.R	MTG.L	0.00010	0.415	0.265	0.362	0.249	0.128	0.272	0.279	0.158	0.141	0.419	**0.402**	**0.001**	P-T
MCC.R	CAL.L	0.00147	0.411	0.342	0.328	0.310	0.127	0.326	0.192	0.338	0.262	0.129	**0.372**	**0.003**	OthF-O
MCC.R	PCUN.R	0.00021	0.734	0.276	0.684	0.237	0.462	0.263	0.296	0.134	0.072	0.681	**0.370**	**0.003**	OthF-P
ROL.R	HES.L	0.00023	0.492	0.250	0.371	0.298	0.198	0.320	0.106	0.599	0.321	0.060	**0.365**	**0.004**	OthF-T
MCC.R	PCUN.L	0.00174	0.665	0.289	0.593	0.246	0.419	0.295	0.306	0.120	0.206	0.236	**0.364**	**0.004**	OthF-P
CUN.L	MTG.L	0.00030	0.338	0.307	0.251	0.267	0.051	0.281	0.082	0.684	0.218	0.209	**0.364**	**0.004**	O-P
MCC.R	SOG.L	0.00091	0.332	0.320	0.222	0.219	0.067	0.276	0.252	0.206	0.226	0.191	**0.362**	**0.004**	OthF-O
IFGoper.L	SMG.R	0.00185	0.659	0.366	0.611	0.232	0.377	0.315	0.101	0.617	0.129	0.460	**0.357**	**0.004**	PreF-P
PCC.R	PCUN.L	0.00043	0.560	0.253	0.441	0.260	0.310	0.268	0.386	0.047	0.239	0.166	**0.349**	**0.005**	P-P
SFGmed.R	ITG.L	0.00134	0.415	0.249	0.313	0.240	0.200	0.249	0.343	0.080	0.270	0.116	**0.347**	**0.006**	PreF-T
MCC.R	HES.L	0.00048	0.345	0.308	0.242	0.292	0.075	0.268	0.110	0.587	0.240	0.165	**0.335**	**0.008**	OthF-T
MCC.L	CUN.R	0.00109	0.438	0.255	0.364	0.226	0.185	0.310	0.360	0.065	0.124	0.478	**0.334**	**0.008**	OthF-O

PCUN.L	PCUN.R	0.00185	1.496	0.288	1.382	0.287	1.248	0.305	0.499	0.008	0.192	0.269	**0.329**	**0.009**	P-P
HES.L	STG.R	0.00092	0.638	0.349	0.455	0.358	0.325	0.350	0.275	0.165	0.314	0.066	**0.324**	**0.010**	T-T
MCC.L	CUN.L	0.00049	0.376	0.294	0.267	0.286	0.097	0.297	0.148	0.461	0.200	0.248	**0.322**	**0.011**	OthF-O
SFGmed.R	MTG.R	0.00009	0.706	0.305	0.534	0.281	0.380	0.302	-0.022	0.915	0.275	0.110	**0.315**	**0.013**	PreF-T
CAL.R	MTG.L	0.00174	0.461	0.299	0.344	0.311	0.191	0.339	0.101	0.615	0.269	0.118	**0.313**	**0.013**	O-P
MCC.R	SOG.L	0.00124	0.342	0.281	0.255	0.220	0.109	0.259	-0.038	0.850	0.205	0.237	**0.307**	**0.015**	OthF-O
MCC.R	CUN.L	0.00009	0.378	0.258	0.251	0.265	0.089	0.276	0.147	0.464	0.143	0.414	**0.300**	**0.018**	OthF-O
SOG.L	MTG.L	0.00113	0.431	0.300	0.325	0.289	0.184	0.265	0.142	0.479	0.182	0.295	**0.286**	**0.024**	O-P
MCC.L	HES.L	0.00094	0.365	0.343	0.218	0.298	0.085	0.289	0.143	0.476	0.205	0.238	**0.280**	**0.027**	OthF-T
HIP.R	CAU.R	0.00012	0.207	0.196	0.094	0.273	-0.048	0.272	0.246	0.216	0.116	0.507	**0.278**	**0.029**	T-B
MCC.R	STG.L	0.00005	0.508	0.385	0.282	0.352	0.129	0.297	0.158	0.432	0.185	0.289	**0.277**	**0.029**	OthF-T
MCC.R	LG.R	0.00019	0.374	0.312	0.246	0.248	0.063	0.300	0.075	0.711	0.071	0.686	**0.274**	**0.031**	OthF-O
CUN.L	MTG.L	0.00095	0.304	0.287	0.225	0.312	0.049	0.285	0.188	0.349	0.066	0.707	**0.267**	**0.036**	O-P
SFG.L	MFG.L	0.00027	0.822	0.275	0.953	0.336	1.131	0.337	0.155	0.440	-0.214	0.218	**-0.264**	**0.038**	PreF-PreF
SMA.R	SOG.L	0.00039	0.346	0.219	0.228	0.208	0.115	0.256	0.214	0.283	0.124	0.477	**0.263**	**0.039**	OthF-O
PHIP.L	SOG.L	0.00022	0.252	0.210	0.141	0.315	0.020	0.244	0.277	0.161	0.134	0.444	**0.261**	**0.041**	T-O
SFGmed.L	MTG.R	0.00067	0.664	0.307	0.492	0.261	0.387	0.297	0.120	0.551	0.217	0.211	**0.261**	**0.041**	PreF-T
LG.R	HES.L	0.00156	0.323	0.259	0.207	0.248	0.084	0.297	0.208	0.299	0.144	0.409	**0.260**	**0.041**	O-T
ROL.R	MCC.R	0.00015	0.447	0.302	0.294	0.301	0.140	0.291	0.089	0.660	0.111	0.525	**0.250**	**0.050**	OthF-OthF
SFGmed.R	CAL.L	0.00127	0.400	0.221	0.244	0.266	0.177	0.282	0.059	0.771	0.297	0.083	0.244	0.056	PreF-O
PHIP.L	SOG.L	0.00043	0.272	0.225	0.158	0.291	0.041	0.254	0.149	0.457	0.144	0.408	0.244	0.056	T-O
MCC.R	STG.R	0.00001	0.621	0.366	0.327	0.339	0.210	0.320	0.079	0.696	0.188	0.279	0.230	0.073	OthF-T
SMA.R	CAL.R	0.00147	0.297	0.210	0.204	0.287	0.079	0.286	0.134	0.506	0.109	0.534	0.229	0.074	OthF-O
MCC.L	STG.L	0.00028	0.545	0.453	0.274	0.379	0.169	0.315	0.169	0.400	0.186	0.285	0.222	0.083	OthF-T
SFGmed.L	PHIP.L	0.00005	0.332	0.261	0.181	0.225	0.034	0.273	0.374	0.054	-0.076	0.663	0.217	0.090	PreF-T

PHIP.L	CUN.R	0.00170	0.298	0.227	0.218	0.271	0.089	0.261	0.297	0.132	-0.007	0.969	0.216	0.091	T-O
HES.L	STG.L	0.00055	0.815	0.311	0.643	0.388	0.501	0.354	0.230	0.248	0.076	0.665	0.211	0.100	T-T
LG.R	MTG.L	0.00166	0.524	0.299	0.375	0.334	0.263	0.316	0.070	0.727	0.123	0.481	0.193	0.133	O-P
PHIP.L	CUN.L	0.00105	0.310	0.242	0.226	0.314	0.094	0.246	0.251	0.207	-0.052	0.766	0.191	0.136	T-O
SFGmed.R	PHIP.L	0.00008	0.278	0.213	0.126	0.202	0.018	0.258	0.293	0.138	-0.024	0.892	0.191	0.137	PreF-T
ROLL	HES.R	0.00124	0.722	0.302	0.488	0.414	0.421	0.378	0.306	0.121	0.163	0.350	0.189	0.141	OthF-T
PCC.L	PCC.R	0.00011	1.141	0.277	0.918	0.315	0.821	0.320	0.441	0.021	0.010	0.955	0.185	0.151	P-P
INS.L	INS.R	0.00088	1.075	0.341	0.845	0.258	0.776	0.329	0.198	0.322	0.114	0.513	0.172	0.182	I-I
MCC.L	STG.R	0.00066	0.603	0.440	0.331	0.320	0.238	0.361	0.187	0.350	0.084	0.629	0.168	0.192	OthF-T
HES.L	HES.R	0.00112	0.802	0.428	0.577	0.433	0.478	0.320	0.079	0.694	0.122	0.485	0.162	0.208	T-T
HIP.L	MTGp.R	0.00160	0.302	0.278	0.143	0.281	0.056	0.301	-0.113	0.573	0.118	0.500	0.149	0.248	T-T
SFGmed.R	MTGp.L	0.00169	0.271	0.252	0.163	0.196	0.081	0.203	-0.050	0.804	0.016	0.927	0.146	0.256	PreF-T
FG.L	SPG.L	0.00108	0.575	0.313	0.400	0.286	0.291	0.330	0.073	0.717	-0.002	0.989	0.135	0.296	O-P
IFGoper.L	STG.L	0.00050	0.556	0.309	0.323	0.261	0.306	0.225	0.018	0.930	0.220	0.204	0.128	0.323	PreF-T
ROLL	HES.L	0.00042	0.709	0.331	0.494	0.417	0.386	0.344	-0.045	0.824	0.058	0.741	0.122	0.346	OthF-T
ROL.R	PoCG.L	0.00094	0.813	0.325	0.556	0.346	0.530	0.311	0.273	0.168	0.097	0.580	0.120	0.353	OthF-P
IFGoper.L	STG.R	0.00128	0.569	0.293	0.349	0.227	0.311	0.302	0.146	0.466	0.058	0.741	0.102	0.431	PreF-T
PHIP.R	PCUN.R	0.00084	0.329	0.242	0.143	0.342	0.122	0.221	0.177	0.378	-0.411	0.014	-0.094	0.466	T-P
PHIP.L	SPG.L	0.00007	0.301	0.230	0.088	0.240	0.018	0.278	0.120	0.550	-0.057	0.744	0.085	0.512	T-P
SMA.L	AMG.R	0.00010	0.425	0.237	0.164	0.301	0.172	0.237	0.129	0.523	-0.226	0.192	-0.084	0.518	OthF-T
Rectus_L	FG.R	0.00090	0.363	0.266	0.167	0.284	0.122	0.271	0.167	0.406	-0.007	0.969	0.083	0.523	PreF-O
PHIP.L	MTG.L	0.00087	0.513	0.281	0.316	0.231	0.253	0.297	0.297	0.133	-0.095	0.587	0.075	0.560	T-T
SFG.R	PHIP.R	0.00083	0.264	0.227	0.111	0.309	0.039	0.265	0.188	0.348	-0.111	0.525	0.074	0.566	PreF-T
PHIP.L	SPG.R	0.00010	0.273	0.221	0.108	0.263	0.032	0.230	0.076	0.706	-0.118	0.499	0.072	0.578	T-P
SFGmed.R	PHIP.R	0.00004	0.287	0.248	0.107	0.224	0.020	0.229	0.236	0.236	-0.228	0.189	0.066	0.612	PreF-T

PHIP.R	ITG.R	0.00024	0.371	0.216	0.135	0.261	0.125	0.267	0.268	0.177	-0.248	0.151	-0.065	0.616	T-T
PHIP.L	PreCUN.R	0.00083	0.409	0.252	0.244	0.316	0.155	0.302	0.305	0.121	-0.201	0.248	0.061	0.640	T-P
AMG.R	PUT.R	0.00053	0.687	0.307	0.461	0.406	0.419	0.270	0.039	0.848	-0.214	0.217	-0.030	0.819	T-B
PHIP.R	PCUN.L	0.00086	0.341	0.268	0.191	0.367	0.118	0.232	0.153	0.445	-0.269	0.118	0.019	0.885	T-P
AMG.R	MTG.L	0.00084	0.371	0.249	0.164	0.257	0.158	0.226	0.069	0.734	-0.083	0.636	-0.017	0.896	T-P
PHIP.R	MOG.R	0.00154	0.341	0.258	0.177	0.279	0.129	0.242	0.095	0.637	-0.198	0.254	-0.006	0.966	T-O
PHIP.L	MTG.L	0.00169	0.316	0.284	0.097	0.284	0.069	0.300	0.085	0.673	-0.088	0.613	0.005	0.970	T-P
PHIP.R	ITG.L	0.00147	0.359	0.232	0.200	0.254	0.116	0.318	0.277	0.161	-0.282	0.101	-0.004	0.974	T-T
AMG.R	MTG.L	0.00174	0.311	0.226	0.152	0.247	0.126	0.216	0.010	0.959	-0.100	0.567	-0.002	0.989	T-P

Note: The abbreviations are listed in Table S2.

Color Fonts mean the strength of the impaired functional connectivity significant correlated with MMSE.

Figure S1.

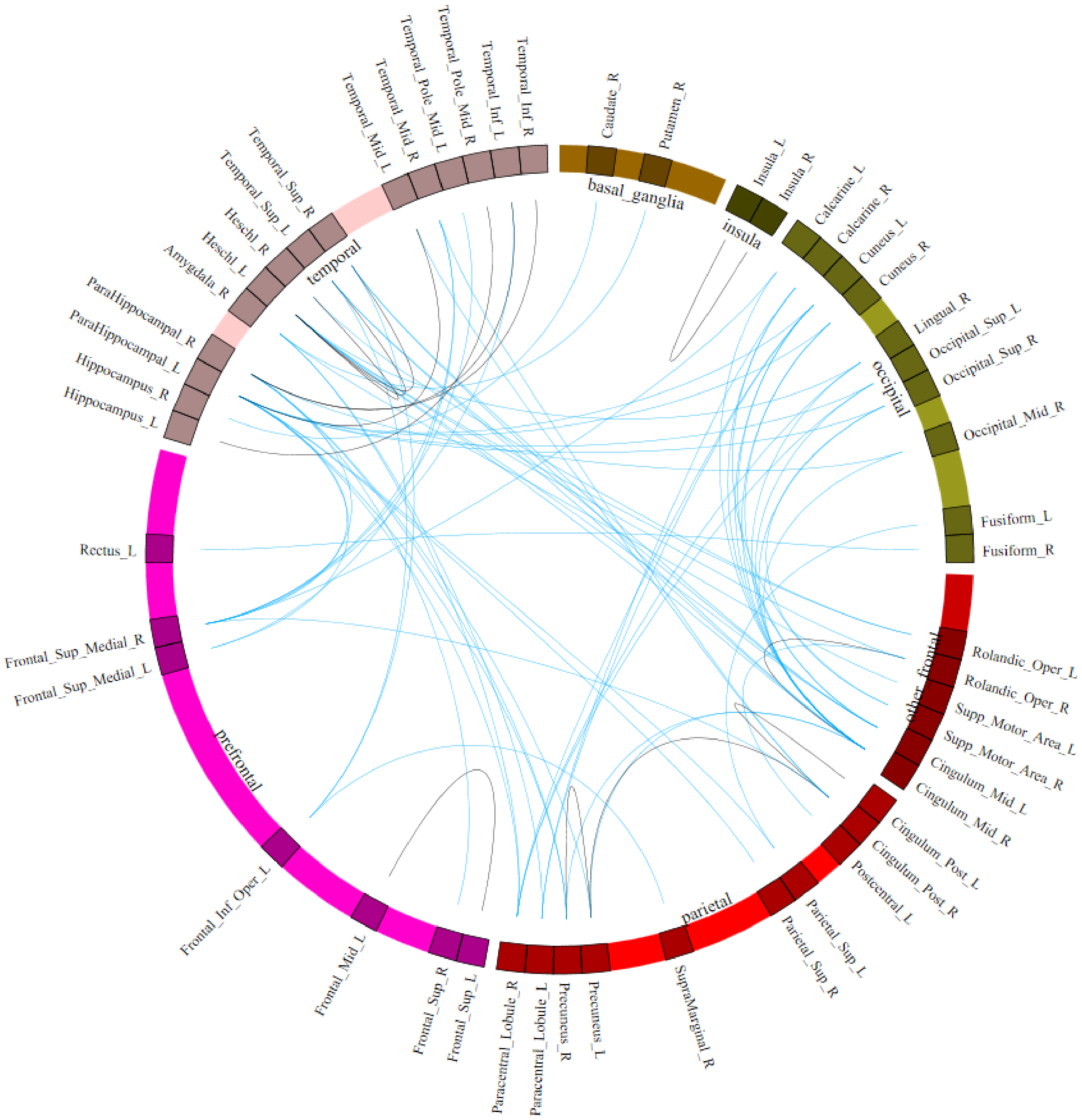

Figure S1. Distributed of the impaired functional connectivity in AD. Each line indicates the mean strength of the functional connectivity between each pair of brain regions. Blue lines represent inter connectivity between lobs and grey lines mean intra connectivity within the defined lobes in Table S2. Detail for these connectivity and it's related to MMSE can be found at Table S3 and Figure 3.

Reference

Liu Y, Yu C, Liang M, Li J, Tian L, Zhou Y, Qin W, Li K, Jiang T (2007) Whole brain functional connectivity in the early blind. Brain 130:2085-2096.

Tzourio-Mazoyer N, Landeau B, Papathanassiou D, Crivello F, Etard O, Delcroix N, Mazoyer B, Joliot M (2002) Automated anatomical labeling of activations in SPM using a macroscopic anatomical parcellation of the MNI MRI single-subject brain. Neuroimage 15:273-289.

Zhang Z, Liu Y, Zhou B, Zheng J, Yao H, An N, Wang P, Guo Y, Dai H, Wang L, Shu S, Zhang X, Jiang T (2014) Altered functional connectivity of the marginal division in Alzheimer's disease. Curr Alzheimer Res 11:145-155.

Zhou B, Liu Y, Zhang Z, An N, Yao H, Wang P, Wang L, Zhang X, Jiang T (2013) Impaired functional connectivity of the thalamus in alzheimer' s disease and mild cognitive impairment: a resting-state FMRI study. Curr Alzheimer Res 10:754-766.
